# Network-Based Relating Pharmacological and Genomic Spaces for Drug Target Identification

**DOI:** 10.1371/journal.pone.0011764

**Published:** 2010-07-26

**Authors:** Shiwen Zhao, Shao Li

**Affiliations:** MOE Key Laboratory of Bioinformatics and Bioinformatics Division, TNLIST/Department of Automation, Tsinghua University, Beijing, China; Deutsches Krebsforschungszentrum, Germany

## Abstract

**Background:**

Identifying drug targets is a critical step in pharmacology. Drug phenotypic and chemical indexes are two important indicators in this field. However, in previous studies, the indexes were always isolated and the candidate proteins were often limited to a small subset of the human genome.

**Methodology/Principal Findings:**

Based on the correlations observed in pharmacological and genomic spaces, we develop a computational framework, drugCIPHER, to infer drug-target interactions in a genome-wide scale. Three linear regression models are proposed, which respectively relate drug therapeutic similarity, chemical similarity and their combination to the relevance of the targets on the basis of a protein-protein interaction network. Typically, the model integrating both drug therapeutic similarity and chemical similarity, drugCIPHER-MS, achieved an area under the Receiver Operating Characteristic (ROC) curve of 0.988 in the training set and 0.935 in the test set. Based on drugCIPHER-MS, a genome-wide map of drug biological fingerprints for 726 drugs is constructed, within which unexpected drug-drug relations emerged in 501 cases, implying possible novel applications or side effects.

**Conclusions/Significance:**

Our findings demonstrate that the integration of phenotypic and chemical indexes in pharmacological space and protein-protein interactions in genomic space can not only speed the genome-wide identification of drug targets but also find new applications for the existing drugs.

## Introduction

Identification of drug targets is one of the major tasks in drug discovery [1]. In recent years, drug phenotypic effects and chemical structures have been used to infer drug-target interactions. Phenotypic effect-based approaches are based on the various phenotypic responses, such as expression profiles and side effects, to external compounds [2]–[5]. Such studies treat the biological system as a whole, and associate one drug to other drugs which have similar biological activity or genes with related phenotypic outcomes. The associated drug pairs are assumed to have the same the targets and the drug-gene pairs are predicted as novel drug-target interactions. On the assumption that structurally similar drugs tend to bind similar proteins, another kind of study using chemical structure-based approaches [6]–[8], especially integrating drug chemical similarity and protein sequence or structure information [9]–[11], has shown lots of encouraging results. These studies also demonstrate that drug chemical structure information is a good indicator for drug biological activity [12].

Though great progress has been made in this field, some challenges still exist. In phenotypic effect-based approaches, similar drug responses may be due to the drugs affecting different targets in the same pathway or in the same biological process, rather than having common targets; also, expression patterns cannot distinguish target genes from downstream regulated genes. Chemical structure-based approaches often focus on a handful of proteins [7], [8], such as those with known interacting drugs [6], [11] or with known three dimensional (3D) structures [9], [10]. For the majority of proteins without such prior information, these approaches are insufficient. Moreover, the underlying assumption in chemical structure-based approaches is not universally true. Examples exist where structurally similar drugs can bind proteins without obvious sequence or structural similarity [13], [14]. Besides, a clear boundary still exists between these two kinds of approaches. Under these circumstances, there is an urgent need to integrate phenotypic and chemical indexes together and develop new methods to predict drug-target interactions on a large scale.

**Figure 1:**
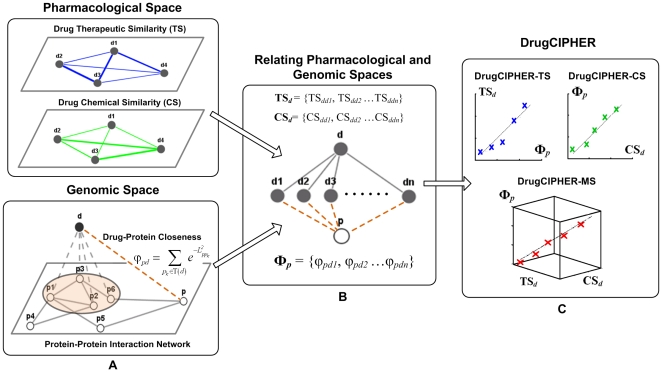
Principle of drugCIPHER. Drugs are solid nodes and presented by ‘*d*’; proteins are hollow nodes and presented by ‘*p*’. **A**). Drug Therapeutic Similarity (TS) (blue solid edges) and Drug Chemical Similarity (CS) (green solid edges) comprise the pharmacological space. The protein-protein interaction (PPI) (gray solid edges) network represents the information in the genomic space. Together with drug-target interactions (gray dashed edges), the closeness (brown dashed edges) is defined to associate a drug with any arbitrary protein. **B**). For drug *d* and protein *p*, two similarity vectors for *d* in pharmacological space (**TS** ***_d_*** and **CS** ***_d_***) and one closeness vector for *p* (**Φ** *_p_*) are constructed. **C**. The concordance scores between drug *d* and protein *p* are computed based on three linear regression models, which assume linear correlations exist between **TS** ***_d_*** and **Φ** *_p_*, **Φ** *_p_* and **CS** ***_d_***, **Φ** *_p_* and the combination of **TS** ***_d_*** and **CS** ***_d_***.

With the development of systems biology and the emergence of chemogenomic approaches, it has been possible to integrate multi-dimensional information and heterogeneous data in drug studies [15]–[17]. Recently, studies found that in pharmacological space, (a) therapeutic similarity (phenotypic index) is, in part, due to the functional relatedness of targets [18], [19], and (b) drugs with similar chemical structure usually bind related proteins [13], [20]; in genomic space, (c) protein (or target) relevance can be characterized by protein-protein interaction (PPI) network features such as modularity or distance [21]. With this understanding, we believe that the similarities in pharmacological space, termed drug Therapeutic Similarity (TS) and drug Chemical Similarity (CS), are correlated with the relatedness of the targets on the basis of the PPI network in genomic space. Based on this assumption, we created a network-based computational framework, drugCIPHER, to relate pharmacological and genomic spaces with multi-dimensional information and predict drug targets on a genome-wide scale (**Figure 1**).

DrugCIPHER takes as input drug TS, drug CS, known drug-target interactions and the PPI network. The TS is established based on the Anatomic Therapeutic Chemical (ATC) classification system [22], [23]. We originally proposed a probabilistic model to characterize the similarity between ATC codes by using a semantic method in machine learning [24], and then to infer the TS. The CS is defined as the 2D structural similarity. Known drug-target interactions and PPI information are obtained from the DrugBank database [25] and the Human Protein Reference Database (HPRD) [26] respectively.

In this work, we first associate a drug and a protein (not necessarily a known target) by defining the ‘closeness’ on the basis of the PPI network. Then, we formulize the previous assumption into three regression models which relate the predefined closeness to TS, CS and the multiple similarity (MS) information combining TS and CS, named drugCIPHER-TS, drugCIPHER-CS and drugCIPHER-MS respectively (**Figure 1**). For a query drug, each protein in the PPI network is assigned three concordance scores based on the different regression models. We did not make a quantitative decision about which protein is the target, as the drug-protein binding affinity itself is a continuous value, not a binary one [14]. Instead, the genome-wide concordance scores describe the importance of the protein to in the activity of the drug, and proteins with large concordance scores could be hypothesized as potential drug targets. As a result, we demonstrate that drugCIPHER-MS outperforms drugCIPHER-TS, drugCIPHER-CS as well as the current Bipartite Local Model (BLM) method [11] in predicting drug-target interactions. Based on drugCIPHER-MS, a genome-wide map of biological fingerprints for 726 drugs is built, and unexpected drug relations, which imply potential novel drug applications and side effects, are generated.

## Results

We extracted 726 Food and Drug Administration (FDA) approved drugs that had at least one known ATC code and known chemical structure information from DrugBank [25] as our reference set. This set was composed of 1176 drug-ATC code interactions and 2225 drug-target interactions. 678 drugs were found with known targets. The human PPI network was retrieved from HPRD [26], and included 38,788 interactions among 9630 proteins. We expanded this network to 9981 proteins by adding, as isolated nodes, 351 target proteins not recorded in the HPRD database. By investigating the relations between drug TS and drug CS, we demonstrated that TS and CS played complementary roles to each other in pharmacological space. The enrichment analysis for drug pairs with common targets with respect to TS and CS was also performed. The results show that drugs with a high TS and CS had a high probability to share targets (**Text S1 and Figure S1**).

### Comparison between pharmacological metrics and genomic metrics

**Figure 2:**
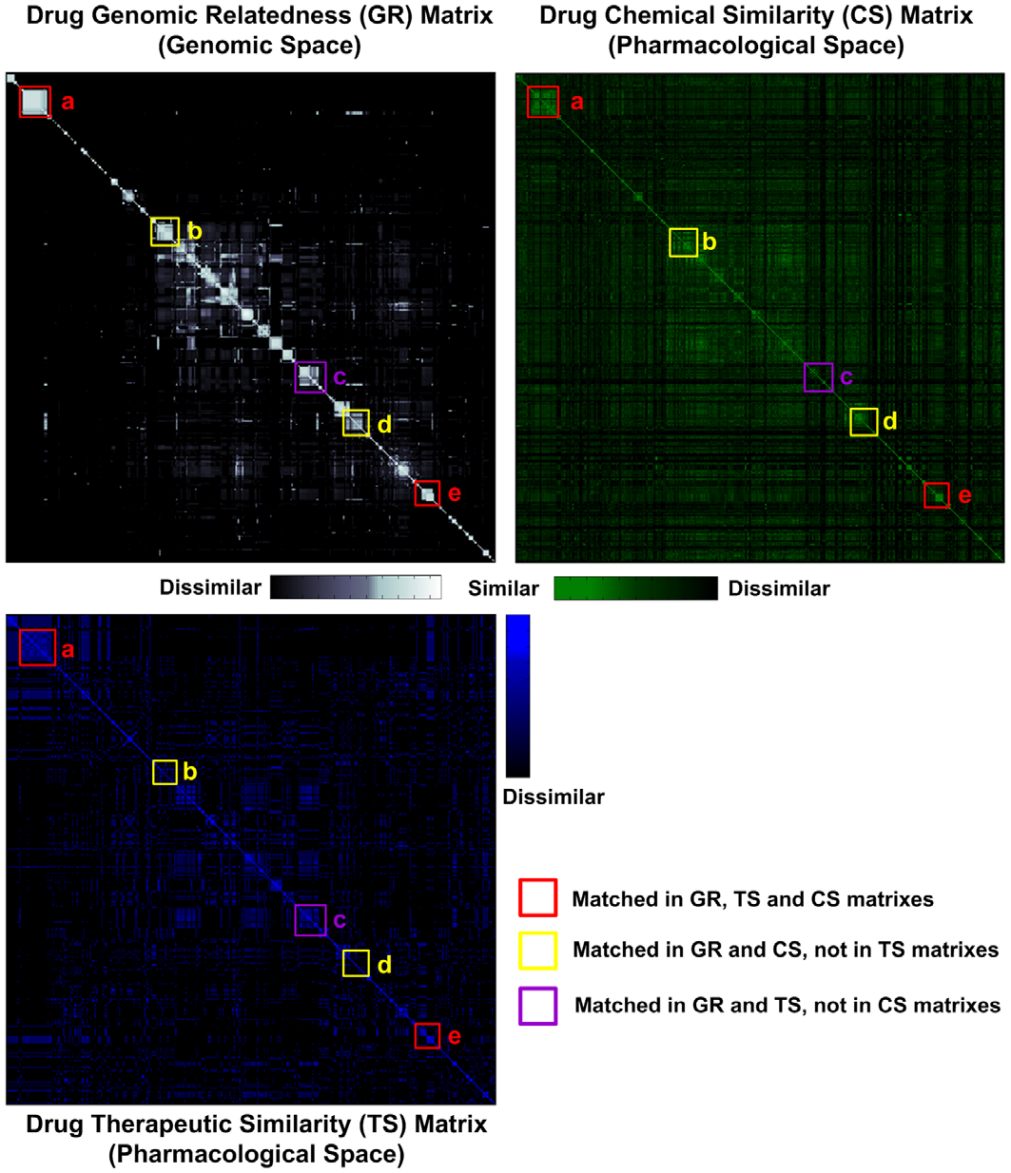
Correlation in pharmacological space and genomic space. Drugs are ordered by clustering their genomic relatedness (GR). Corresponding TS and CS matrixes are aligned next to the GR matrix, and all of them are demonstrated by heat maps. Modest but significant correlations are observed between pharmacological similarities and genomic relatedness (P<0.0001).

As a step toward drugCIPHER, we investigated the relations between drug similarities in the pharmacological space and drug genomic relatedness (GR) in the genomic space, where GR is defined as the average closeness of drug targets in the PPI network (**See** **Materials and Methods**). The similarity matrixes for TS, CS and GR are shown in **Figure 2**. Drugs are ordered by clustering of their GR for observation. In the GR matrix, we observe many small blocks enriched in the diagonal, indicating the targets of these drugs were strongly related in the PPI network. Some blocks can be matched in both the CS matrix and the TS matrix (**block a and e**), suggesting a consistency between the two spaces. There are also some blocks with no similar patterns in other matrixes (**block b, c and d**). These phenomena show that drugs with high genomic relatedness and chemical similarity may generate different therapeutic effects (**block b and d**), and drugs with diverse structures could still have a similar therapeutic activity and related targets (**block c**).

To quantify the correlations between TS, CS and GR, we computed Spearman correlation coefficient between GR and the corresponding TS and CS. The correlation coefficients are 0.0957 for GR and TS and 0.1465 for GR and CS, indicating that each has a slight positive correlation. We randomly shuffled the drug labels 10,000 times to evaluate the significance of such correlations. The results suggest that correlations between TS, CS and GR are about 2.2 and 1.5 fold of the maximum permuted coefficients, demonstrating that such modest correlations are still significant (P<0.0001) (**Text S1, Figure S2**).

### Performance of drugCIPHER

**Figure 3:**
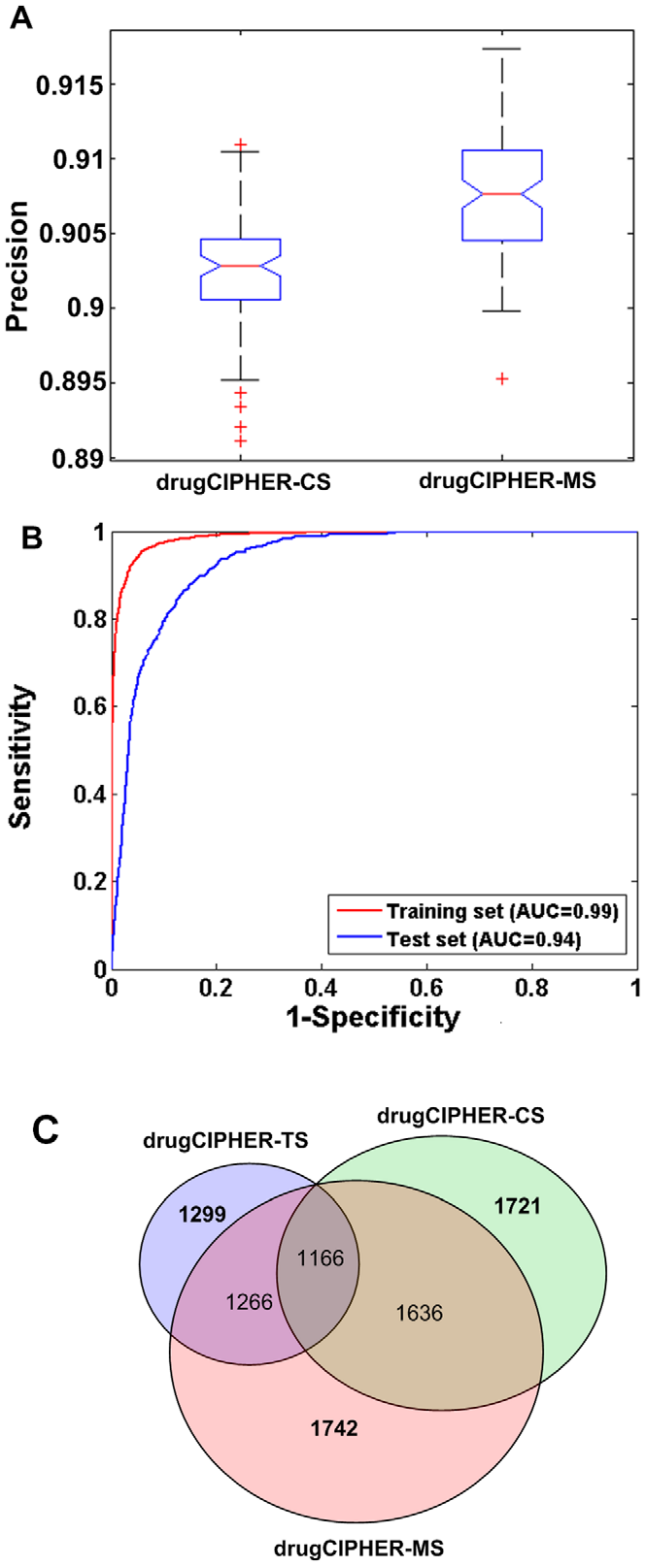
Performance of drugCIPHER. **A**). Comparison between drugCIPHER-CS and drugCIPHER-MS in leave-one-out cross-validation. The outliers are defined as the points larger than q_3_+1.5*(q_3_−q_1_) or smaller than q_1_−1.5*(q_3_−q_1_), in which q_1_ and q_3_ are the 25^th^ and 75^th^ percentiles, respectively. **B**). ROC curves of drugCIPHER-MS for the training set and the test set. The AUC is 0.988 for the training set, and 0.935 for the test set. **C**). The constitution of known drug-target interactions ranked in the top 100 by drugCIPHER-TS, drugCIPHER-CS and drugCIPHER-MS.

We proposed a novel method, drugCIPHER, to relate pharmacological and genomic spaces, and demonstrated the good performance of this method in recovering known drug-target interactions in DrugBank by using leave-one-out cross-validation. For each known drug-target interaction, 19 negative controls from the 9981 proteins in the PPI network were added, forming a candidate set. To simulate the prediction of unknown targets, we equated this process to remove all targets except one (**See** **Materials and Methods**). The three models of drugCIPHER were employed to prioritize the proteins in the candidate set. We defined a success if the known target was ranked at the top, and the precision as the proportion of successes after running drugCIPHER on all known drug-target interactions. After 100 repeats, on average, drugCIPHER-TS, drugCIPHER-CS and drugCIPHER-MS get precisions of 0.783, 0.903 and 0.908 respectively (**Table 1**). The results show that the performance of drugCIPHER-MS is not only much better than drugCIPHER-TS but also better than drugCIPHER-CS with statistical significance (P = 7.94e-015, Wilcoxon rank sum test) (**Figure 3A**).

**Table 1:** Performance comparison of drugCIPHER-TS, drugCIPHER-CS and drugCIPHER-MS.

drugCIPHER	TS	CS	MS
Validation procedure (precision)	0.783	0.903	0.908
Training set (AUC)	0.964	0.981	0.988
Test set (AUC)	0.849	0.917	0.935

Then, based on the known drug-target interactions in DrugBank, we applied drugCIPHER to the 726 FDA approved drugs in the reference set and the 9981 proteins in the PPI network to give a genome-wide inference of drug-target interactions. Known drug-target interactions were used as golden standards to evaluate the overall performance of drugCIPHER. We ranked the 9981 proteins according to the concordance score for the 678 known-target drugs. Proteins above a given rank threshold were treated as predicted targets (positives), and the rest were viewed as non-targets (negatives). Following this principle, sensitivity and specificity could be defined. The results show the Area Under the ROC Curve (AUC) for drugCIPHER-MS reaches 0.988 (**Figure 3B**), and for drugCIPHER-TS and drugCIPHER-CS the values are 0.964 and 0.981 respectively (**Table 1**). For example, when we set the rank threshold to 100, 1299 out of 2225 known drug-target interactions (58.4%) are successfully identified by drugCIPHER-TS, and 1721 (77.3%) are identified by drugCIPHER-CS; 1166 (52.4%) are identified by both of the models (**Figure 3C**). Moreover, the 1166 interactions are all ranked above the given threshold by drugCIPHER-MS, which in total identifies 1742 (78.3%) known drug-target interactions above this threshold (**Figure 3C**).

We further introduced an independent data set to test the generalization ability of drugCIPHER. We extracted drug-protein binding information from the Psychoactive Drug Screening Program (PDSP) Ki database [27]. Interactions with a Ki binding affinity lower than 10 µM were viewed as drug-target interactions [5]. We eliminated the interactions which have already been recorded in DrugBank. 513 additional drug-target interactions were found. Using the previous rank lists, we computed the ROC curves for the additional interactions. An AUC of 0.935 for drugCIPHER-MS is observed (**Figure 3B**), whereas drugCIPHER-TS and drugCIPHER-CS have an AUC of 0.849 and 0.917 respectively (**Table 1**), indicating the drugCIPHER models do not overfit the data.

To give an illustration of the best model, drugCIPHER-MS, we investigated Oxytocin, Nefazodone and their targets. Oxytocin is famous for its pleiotropic activities including induction of labor and influences on social behaviors [28]. As shown in **Table 2**, two targets of Oxytocin recorded in DrugBank are ranked 1^st^ and 2^nd^ by drugCIPHER-MS. Additionally, we find 4 proteins with a Ki lower than 10µM in the PDSP Ki database. Without prior knowledge, drugCIPHER-MS ranks them at 3^rd^, 47^th^, 48^th^ and 91^st^ out of 9981 possibilities. For Nefazodone, an antidepressant therapy [29], all 5 of the targets in DrugBank are ranked in the top 3% by drugCIPHER-MS, generating a ∼33 fold enrichment (P = 4.9e-6, Fisher exact test, one sided). Three additional drug-target binding interactions are identified in the PDSP Ki database, all of which are ranked above 120^th^ (1.2%), with a ∼84 fold enrichment (P = 3.1e-5, Fisher exact test, one sided) (**Table 2**). It should be noted that other high-ranking proteins may also be of interest and may be indicative of potential off-target effects.

**Table 2:** Ranks of known targets (DrugBank) and binding proteins (PDSP database) for Oxytocin and Nefazodone.

Drug	Database	drugCIPHER-MS Rank	Target Gene Symbol	Entrez ID	Ki
Oxytocin	DrugBank	1	PREP	5550	
		2	OXT	5020	
	PDSP	3	OXTR	5021	0.5nM
		47	AVPR1B	553	1782nM
		48	AVPR2	554	1544nM
		91	AVPR1A	552	123nM
Nefazodone	DrugBank	9	HTR2A	3356	
		12	SLC6A4	6532	
		33	SLC6A2	6530	
		267	ADRA1B	147	
		305	ADRA1A	148	
	PDSP	32	DRD2	1813	910 nM
		103	SLC6A3	6531	360 nM
		119	HTR1A	3350	80 nM

### Comparison with other methods

Previously, related studies which focused on a limited number of proteins [6]–[8], [11] suffered from limitations in high-throughput discovery of new drug-target interactions. To the best of our knowledge, though target identification on a genome-wide scale has been performed [3], there are no quantitative results we can compare with. Thus, we only try to compare drugCIPHER with a currently available non-genome-wide method, the BLM [11], which is also the most precise model for target prediction. We find that the AUCs in the BLM achieve 0.973, 0.970, 0.953 and 0.858 for four drug sets: drugs targeting enzymes, ion channels, G protein-coupled receptors and nuclear receptors with known drug-target interactions of 2926, 1476, 635 and 90 respectively. We averaged the performance of the BLM by the weights of the number of corresponding interactions, generating an AUC of 0.9676. As shown in **Figure 3B** and **Table 1**, both drugCIPHER-CS (AUC = 0.981) and drugCIPHER-MS (AUC = 0.988) have better performances. Moreover, there is no clear result about the generality of the BLM. In contrast, the generality of drugCIPHER-MS is well demonstrated.

### A genome-wide map of drug biological fingerprints

The genome-wide concordance scores produced by drugCIPHER-MS implied the importance of each protein in the biological activity of a given drug, therefore they can be viewed as a drugs biological fingerprint. We eliminated unspecific proteins which always received consistent scores for the 726 drugs, leaving 9639 proteins (**Text S1, Figure S3A**). A genome-wide map of predicted biological fingerprints is comprised of the 9639 concordance scores (http://bioinfo.au.tsinghua.edu.cn/drugCIPHER/Drug_biological_fingerprints.rar). We find the predicted fingerprint a better indicator for identification of drug targets compared to the therapeutic index and chemical structure, which merely include information in pharmacological space (**Text S1, Figure S3B**). A two-way hierarchical clustering for the 726 biological fingerprints was also performed to explore the global drug-target (protein) interactions (**Text S1, Figure S4**).

### Potential novel drug applications and side effects

**Figure 4:**
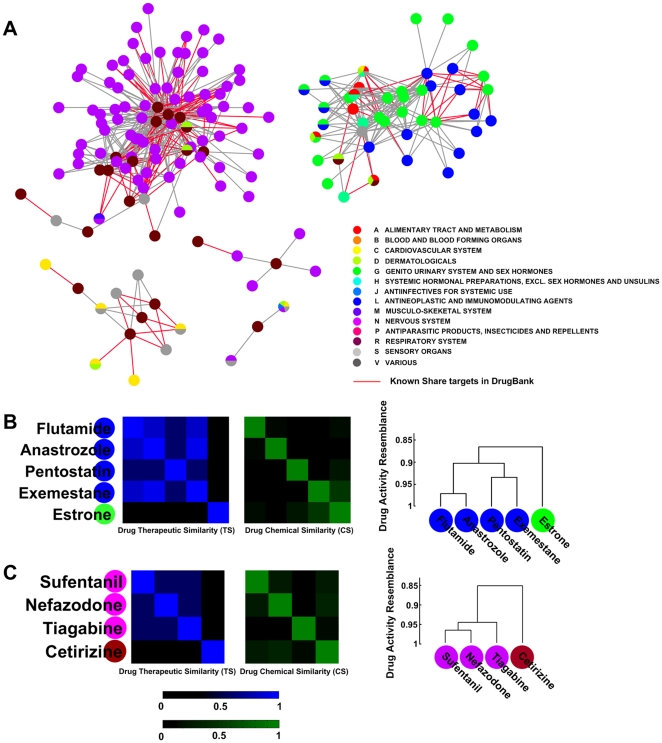
Exploration of novel drug applications and side effects. **A**). Unexpected drug relations less than the significance level of 0.05, including 158 drugs and 501 relations. Drugs are colored according to their first level of ATC code. Drug pairs with known common targets are highlighted by red edges. **B**). Estrone and the corresponding cluster. Four antineoplastic drugs are associated with Estrone, a hormonal therapy (P<0.05). From small to large, the linkage resemblances (averaged) are 0.86, 0.90, 0.93, and 0.97 in this cluster. **C**). Cetirizine and the corresponding cluster. Three nervous system related-drugs are associated with Cetirizine, an anti-allergic therapy (P<0.05). The linkage resemblances (averaged) in this cluster are 0.85, 0.95, and 0.97 respectively.

We further define the drug activity resemblance as the cosine of the drug biological fingerprints and find the fingerprints can provide an alternative way to discover new drug applications and side effects. We find that some drugs, though with different main ATC categories, have similar biological fingerprints and are clustered tightly in the hierarchical clustering. Such drug pairs with an activity resemblance less than the significance level of 0.05 (resemblance = 0.84) were extracted (**Figure 4A**, **Table S1**), including 501 unexpected relations among 158 drugs.

Drug pairs with no clear chemical similarity and no common targets were extracted, as none of these interactions is obviously predictable using current knowledge. For example, Estrone, an estrogen classified as ‘G’ in the ATC main category, is closely associated with four antineoplastic drugs classified as ‘L’ in the ATC main category (P<0.05) (**Figure 4B**). Typically, Estrone is connected with Exemestane (an Aromatase inhibitor, that disrupts the synthesis of estrogens and is used to treat various cancers [30]) with an activity resemblance of 0.906 (P = 0.024). Interestingly, although Estrone and the drugs it clusters with have different therapeutic effects and dissimilar chemical structures (maximum TS = 0 and CS = 0.4) and although they do not have any known common targets, the apoptotic action of Estrone has already been discovered, which makes it a promising antineoplastic agent [31], [32]. DrugCIPHER-MS successfully predicted this novel application. Another example is Cetirizine, an anti-histamine agent used as an anti-allergic therapy [33] (**Figure 4C**), which was connected with three nervous system related-drugs (P<0.05). Similarly, no significant TS or CS is found (the maximum TS and CS are 0 and 0.5), and no common target between Cetirizine and other drugs has been identified. Nevertheless, the side effects of Cetirizine on the nervous system have been reported [34] and supported by the SIDER database [35] (**Text S1**). DrugCIPHER-MS also successfully detected these unexpected interactions.

## Discussion

In this study, by relating pharmacological space with genomic space on the basis of the PPI network, drugCIPHER successfully identified drug-target interactions and predicted biological fingerprints *in silico* for 726 FDA approved drugs. Previously, drug biological profiles have been addressed by experimental approaches or computational methods [2], [4], [16], [36]. Alternatively, we presented another way to generate such profiles (biological fingerprints) and provided an interesting perspective for understanding drug activity. More importantly, our methods extend the candidate target proteins to a genome-wide scale (9981 proteins), which greatly enlarges the number of known targets (935 proteins) in DrugBank. Owing to the fact that every protein could be susceptible to drugs, this preliminary study provides us with valuable clues for identification of drug-target interactions on a large scale.

The success of drugCIPHER-MS can be attributed to a number of aspects. First and most importantly, the two complementary indexes, therapeutic activity and chemical structure, are integrated together in this model, enabling us to capture compound activity comprehensively and bolster the efficiency of target identification. Second, our method benefits from current knowledge such as the known drug-target interactions, which provide us with golden standards for understanding drug mechanisms. Third, topological properties in the PPI network reflect certain basic characteristics of biological systems. Together with known drug-target interactions, such information makes it possible to relate pharmacological space with genomic space. Thus, we believe that combining heterogeneous information could help to generate new hypotheses and boost further drug discovery.

Based on drugCIPHER-MS, a genome-wide map of drug biological fingerprints for 726 drugs was predicted. One aspect of the results merits emphasis. By integrating TS and CS in pharmacological space and PPIs in genomic space, unexpected drug relations emerge, which demonstrate that the integration of existing multi-dimensional information may generate additional knowledge. At a significance level of 0.05 of the activity resemblance, 501 unexpected drug-drug relations are obtained (**Table S1**). Nevertheless, drug pairs with an activity resemblance smaller than 0.84 may still present pharmacological meaning. As shown in **Figure S5**, the blocks in the activity matrix which are not present in the TS matrix may indicate new drug applications or side effects (**Text S1, Table S2**).

With the development of pharmacology, more and more attention has been paid to chemogenomics [15], a discipline that tries to understand the global effects of a compound in a complete biological system. Analogous to reverse and forward principles in chemogenomics, two primary applications of the biological fingerprints can be found. (a) Reverse applications: when a new gene of interest is identified, one could quickly aim at a handful of candidate drugs which are most relevant to this gene, therefore effectively narrowing down the entire compound library and increasing the efficiency of high-throughput screening in drug discovery. (b) Forward applications: the biological fingerprints are predicted on the basis of the whole biological system. To identify new drug targets, one can select the top ranked proteins in the fingerprints, and design experiments to validate these proteins, such as docking or *in vitro* binding assays. Together with other experimental data [4], [36], these biological fingerprints allow us to identify drug targets more quickly and confidently.

Currently, there are still some limitations in our methods. First, our methods are limited to a part of the entire genome: proteins with known PPIs. Therefore the completeness and quality of PPIs influence the results. As we used the gene name to represent the protein, the gene-protein discordance caused by events such as alternative splicing is currently not considered. Our future work will address the variations in the protein structure brought about by alternative splicing and its effects on drug-target interaction patterns as well as drug biological activities. Second, we assume each protein has the potential to bind small molecules. Actually, more aspects should be considered such as the druggability, cellular compartmentalization and protein level. Third, in our models, some prior knowledge about the drugs is needed, e.g. the chemical structures and the ATC codes. As the chemical structure information has been extensively addressed, we can use drugCIPHER-CS instead of drugCIPHER-MS to enlarge the reference set while sacrificing some precision. It must be noted that the ATC classification system is not the only way to address the drug therapeutic similarity. Alternatives include pharmacology annotations or clinical records.

In summary, this work demonstrates that the integration of multi-dimensional information in pharmacological space and genomic space gains advantages in target identification and yields additional knowledge. More importantly, the global concordance score presents a novel understanding of drug-protein interactions, and the predicted biological fingerprints could also provide us new insights into associating drugs with diseases and pathways, predicting new drug applications, as well as deciphering drug side effects. Together with network pharmacology [37], this preliminary study is one step toward genome-wide drug target identification.

## Materials and Methods

### Data sources

**Figure 5:**
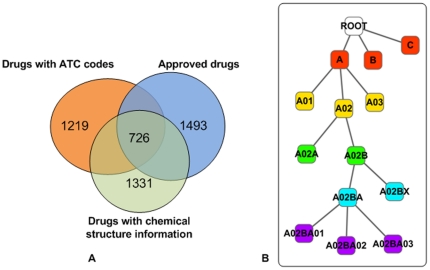
Data sources and the ATC classification system. **A**). The constitution of the reference set. **B**). The sketch of the hierarchical structure of the ATC classification system and ATC codes. The leaf nodes represent the ATC codes.

The drug-ATC code interactions and known drug-target interactions were obtained from DrugBank [25] in January 2010. We extracted drugs which were (a) FDA approved, (b) with at least one ATC code and (c) with chemical structure information recorded in the KEGG compound database [38]. 726 drugs were obtained (**Figure 5A**), together with 1176 drug-ATC code interactions. Targets which were DNA or small RNAs were removed, as we only considered interactions between drugs and proteins, generating 2225 drug-target interactions for 678 drugs.

Protein-protein interaction information was retrieved from HPRD [26] in January 2010. 38,788 interactions among 9630 human proteins were obtained. 351 target proteins absent in the interactome were added into the PPI network as isolated nodes, expanding the network to 9981 proteins.

Drug-protein binding interactions were retrieved from the PDSP Ki database [27] in February 2010. Interactions with a Ki binding affinity lower than 10µM were viewed as drug-target interactions [5]. We eliminated the interactions which have already been included in DrugBank to make the training set and test set independent of each other. After mapping this data to our reference set, we found 513 additional drug-target interactions for 86 drugs.

### Drug therapeutic similarity (TS) and chemical similarity (CS)

The drug (TS) was addressed based on the similarity of ATC codes (**Figure 5B**) by proposing a probabilistic model [24]. The similarity between two ATC codes is derived according to their prior probabilities (frequency) and the probability of their commonality, which is defined as their longest matched prefix:
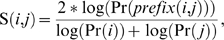
(1)where *prefix(i,j)* is the longest matched prefix of ATC code *i* and *j*. Note that drugs may have more than one ATC code, we define the maximum ATC code similarity as TS:
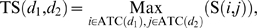
(2)where ATC(*d*) represents all the ATC codes belonging to drug *d*.

The drug CS was computed based on the Tanimoto coefficient [39].

### Drug-protein closeness and drug genomic relatedness (GR)

We associate pharmacological space with genomic space by defining the closeness between a protein *p* and a drug *d* on the basis of the PPI network:
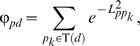
(3)where *p_k_* is the known target of the given drug *d*. *L_ppk_* is the shortest distance between *p* and *p_k_* in the PPI network. 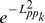 is used to convert protein-protein distance to protein-protein closeness. This equation denotes that the closeness between drug *d* and protein *p* equals the summation of closeness between *p* and all targets of *d*. If two proteins are disconnected, we define *L_ppk_* = ∞.

Then, given drugs *d_1_* and *d_2_*, we define the drug GR as the averaged closeness among their known targets:
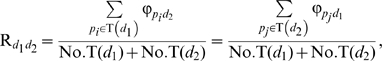
(4)where No.T(*d*) represents the count of known targets belonging to drug *d*.

### DrugCIPHER

Previously, by integrating phenotypic similarity and the PPI network, we successfully proposed a model named CIPHER to infer disease-gene relations [40]. Here, we extrapolate this idea to predict drug-target interactions and call the current framework drugCIPHER, named after CIPHER.

### DrugCIPHER-TS

We assume the relevance in genomic space is responsible for drug TS. With equation (3), given two drugs *d* and *d_j_*, we formulize this assumption into the following equation:
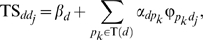
(5)where *p_k_* is the known target of drug *d*. Equation (5) denotes that the TS between *d* and *d_j_* can be described as the linear combination of closeness between drug *d* and all the targets belonging to drug *d_j_*. *β_d_* and *α_dpk_* can be interpreted as some constants.

Then, we define the similarity vector between drug *d* and all n drugs as **TS** ***_d_*** = {TS*_dd1_*, TS*_dd2_* …TS*_ddn_*} and the closeness vector between protein *p* and n drugs as **Φ** ***_p_*** = {ϕ*_pd1_*, ϕ*_pd2_* …ϕ*_pdn_*}, and expand equation (5) to
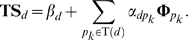
(6)The concordance score between drug *d* and protein *p* in drugCIPHER-TS is defined as
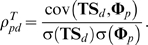
(7)This concordance score describes the degree of contribution of protein *p* to the TS vector of drug *d* in equation (6), therefore it is viewed as the potential likelihood of protein *p* being targeted by drug *d*.

### DrugCIPHER-CS

In this model, we believe the closeness between drug *d* and protein *p* can be explained by the drug chemical similarity (CS), and formulize such a consideration as follows:
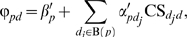
(8)where *d_j_* is the known drug binding to the given protein *p*. Equation (8) suggests the closeness between drug *d* and protein *p* can be described as a linear combination of the chemical similarities between *d* and all the drugs binding to *p*. This equation also echoes the Similarity Ensemble Approach (SEA) principle [13], [14]. Similarly, *β′_p_* and *α′_pdj_* can be treated as some constants.

Correspondingly, we define the similarity vector **CS** ***_d_*** for drug *d* as {CS*_dd1_*, CS*_dd2_* …CS*_ddn_*}, and extend equation (8) into
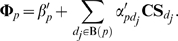
(9)We define the concordance score in drugCIPHER-CS as
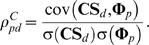
(10)This concordance score describes the degree of the contribution of drug *d* to the closeness vector **Φ** ***_p_*** of protein *p* in equation (9), therefore it is treated as the likelihood of drug *d* targeting protein *p*.

### DrugCIPHER-MS

In this model, we integrate TS and CS and propose a multiple-similarity based regression model. Given protein *p*, we consider both equations (6) and (9) and assume:
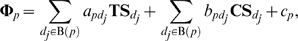
(11)where *a_pdj_*, *b_pdj_* and *c_p_* are some constants. To simplify equation (11), we generally believe drug *d* will mostly contribute to (11) when it maximally fits the following equation:
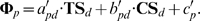
(12)We first estimate *a′_pd_* and *b′_pd_* by least-square solutions, 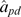 and 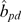, and then define the concordance score in drugCIPHER-MS as
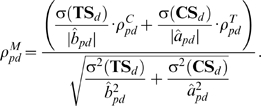
(13)This concordance score describes the degree of fitness of drug *d* for the closeness vector of protein *p* (**Φ** ***_p_***) considering both TS and CS. The larger the concordance score is, the more important role *p* plays in the biological activity of *d*, and the more likely it is that *p* is the target of *d*.

### Validation procedure

In leave-one-out cross-validation, for each drug-target interaction, 19 negative control proteins and the positive target composed the validation set. The negative control proteins were randomly chosen from the whole PPI network with equal probability. To simulate the prediction of unknown targets, we equated this process to remove all targets except the positive one. According to equation (3), the closeness between the proteins in the validation set and the drug therefore must be modified. Here, we subtracted the closeness of these proteins to the removed targets from the closeness of the proteins to this drug, which was equivalent to recalculate the drug-protein closeness by taking these removed targets as unknown targets.

## Supporting Information

Text S1 Preliminary investigations and additional results.
(0.05 MB DOC)

Table S1 501 unexpected drug-drug relations with corresponding ATC codes, activity resemblance and significance level.
(0.49 MB XLS)

Table S2 Drug indexes for the comparison of drug therapeutic similarity and activity resemblance.
(0.06 MB XLS)

Figure S1 (a) Relationship between drug therapeutic similarity and chemical similarity. (b) Smoothed associations between drug therapeutic similarity and chemical similarity. (c) Fold enrichment analysis of therapeutic similarity and chemical similarity with respect to common target drug pairs.
(10.15 MB TIF)

Figure S2 (a) Permuted correlation coeffecients for therapeutic similarity and dug genomic relatedness. (b) Permuted correlation coefficients for chemical similarity and drug genomic relatedness.
(7.95 MB TIF)

Figure S3 (a) The GO annotations (cellular component) for eliminated proteins. (b) Precision-Recall curves in recovering drug pairs with common targets.
(6.97 MB TIF)

Figure S4 Two-way cluster for drug biological fingerprints.
(9.62 MB TIF)

Figure S5 Comparison of drug therapeutic similarity and activity resemblance for unexpected drug-drug relations.
(3.75 MB TIF)
