# Supplementary material for: Network-Based Relating Pharmacological and Genomic Spaces for Drug Target Identification
Source: PLoS One. 2010 Jul 26;5(7):e11764. doi: 10.1371/journal.pone.0011764 (PMC2909904; doi:10.1371/journal.pone.0011764)
Supplement: Text S1 — Preliminary investigations and additional results. (0.05 MB DOC) [file pone.0011764.s001.doc]

## Supporting text S1

Network-based Relating Pharmacological and Genomic Spaces for Drug Target Identification

Shiwen Zhao and Shao Li*

* Email: [shaoli@mail.tsinghua.edu.cn](mailto:shaoli@mail.tsinghua.edu.cn)

MOE Key Laboratory of Bioinformatics and Bioinformatics Division, TNLIST / Department of Automation, Tsinghua University, Beijing, China

## Contents:

## Preliminary Investigations

Relation between drug therapeutic similarity and chemical similarity

Enrichment analysis for drug pairs with common targets

## Additional Results

Permutations for pharmacological and genomic metrics

Elimination of unspecific proteins

Evaluation of therapeutic index, chemical structure and biological activity resemblance

Two-way hierarchical cluster

Side effect of Cetirizine

Exploration of unexpected drug-drug relations

## References

## Preliminary Investigations

## Relation between drug therapeutic similarity (TS) and chemical similarity (CS)

TS was computed based on the ATC classification system [1], which partially includes drug chemical information. However, this information only describes a sketch of the chemical category, and it does not contain details such as molecular structure.

To explore the relation between TS and CS, we compared the CS scores with the TS scores between each drug pair in our reference set **(Figure S1A)**. To make the relationship more clear, we smoothed the result as follows. First, the similarity score pairs were sorted according to the CS. Then, a window of size 500 was used to smooth the sorted score pairs. We averaged the CS scores in the window as well as the corresponding TS scores. After the window traversed through all the pairs by step 50, the smoothed relation was generated **(Figure S1B)**.

We find some drug pairs, though with a high TS score, are distinct in chemical structure, and vice verse. For example, Aluminium and Benzocaine share an ATC code of A01AD11, generating a TS of 1, whereas their CS is 0. Another example is Bismuth and Lithium. They have a CS of 1, however, their TS is 0, indicating their ATC codes are different from the first level in the ATC classification system.

## Enrichment analysis for drug pairs with common targets

It is hoped that drug pairs with higher similarity are more likely to share targets. To address this question, we investigated the enrichment of drug pairs with common targets with respect to their TS and CS.

In our reference set, there are 6801 drug pairs with common targets, implying a proportion of $6801/\left(726\text{ }choose\text{ }2\right)=0.0258$. We sorted drug pairs according to their similarity in a descending order. Given a similarity threshold, we computed the proportion of drug pairs with common targets above this threshold. The fold enrichment was defined as the ratio of the two proportions. For example, when setting the similarity threshold to 0.5, the proportion of drug pairs with common targets above this threshold is 0.5592, generating a fold enrichment of $0.5592/0.0258=21.67$. We investigated the fold enrichments for CS and TS with respect to different similarity thresholds. The results are demonstrated in **Figure S1C**. Note that for the two similarities, the same threshold score represents different meanings, therefore they should be treated separately rather than comparatively. We find the maximum of fold enrichment of TS is 25.8 with the threshold of 0.95. For CS, the maximum of fold enrichment is not accompanied with the highest similarity score: the fold enrichment reaches 29.4 with a threshold of 0.75.

## Additional Results

## Permutations for pharmacological and genomic metrics

To examine the significance of the Spearman correlations between pharmacological metrics and genomic metrics, we randomly permuted the drug labels in the TS and CS metrics and then computed the respective Spearman correlation coefficients with the drug genomic relatedness (GR). The 10,000 permuted coefficients are shown in **Figure S2A** and **S2B**. The results suggest that correlations between TS, CS and GR are significant (P<0.0001), with about 2.2 and 1.5 fold of the maximums of the permuted coefficients.

## Elimination of unspecific proteins

For further analysis, we excluded the proteins which were assigned consistent concordance scores for all drugs in drugCIPHER-MS. 342 proteins are excluded, and none of them is a known drug target. We analyzed these proteins on the basis of the PPI network. None of the 342 proteins is connected to the largest component in the PPI network; they form either isolated nodes or small sub-clusters apart from the giant component. The GO annotations (cellular component) for these proteins are shown in **Figure S3A**.

## Evaluation of therapeutic index, chemical structure and biological activity resemblance

It is hoped that the predicted fingerprints could be a better indicator for drug target identification compared with the therapeutic index and chemical structure, which merely include information in pharmacological space. To explore this consideration, we defined the drug biological activity resemblance as the cosine of the include angle of the biological fingerprint vector. We evaluated the performance of TS, CS and the activity resemblance in recovering drug pairs with known common targets. We ranked drug pairs with respect to TS, CS and activity resemblance. Given a similarity threshold, we computed the proportion of drug pairs with common targets above this threshold and defined such a proportion as the precision. Correspondingly, we defined the recall as the proportion of drug pairs known to share targets above the threshold to all drug pairs with common targets in our reference set. With different thresholds, the Precision-Recall curves for TS, CS and activity resemblance are computed (**Figure S3B**). With a decrease of the threshold, the precision decreases and the recall increases correspondingly. As we expected, the areas under the curve are 0.18, 0.23 and 0.27 respectively for TS, CS and activity resemblance, suggesting that the biological fingerprints have a better performance in recovering drug pairs with common targets. Typically, for activity resemblance, when setting the threshold to 0.945, a >50% precision with a >20% recall is observed, generating a ~20 fold enrichment of true positives.

## Two-way hierarchical cluster

A two-way hierarchical clustering was performed to explore the drug-target (protein) interactions globally (**Figure S4**). Drugs were clustered according to their similar biological fingerprints, and proteins were clustered based on the overlaps of the related drugs. Drug clusters were annotated with the ATC main categories. There are some drugs with more than one ATC main category. Such additional categories were annotated in parallel. Protein clusters were annotated by their enriched GO terms (biological process). The modularity of drug-protein relations emerges in the two-way hierarchical clustering. For example, in the highlighted module, nervous system therapies are related to proteins enriched with the cell-cell signaling biological process. Note that drugs may relate to multiple protein clusters, which might indicate multiple mechanisms of action and potential polypharmacology, and proteins may relate to multiple drug clusters, which suggest their promiscuities.

## Side effect of Cetirizine

In the SIDER database [2], the side effect ‘Drowsiness’ was associated with Cetirizine. Six recorded frequencies of occurrence of ‘Drowsiness’ in the drug treatment (case) were 1.3%, 1.9%, 2.88%, 4.2%, 5.23% and 5.7%, and four frequencies in the placebo treatment (control) were 0.417%, 1.3%, 1.75% and 1.9%. The results suggest that the association between ‘Drowsiness’ and Cetirizine is significant (P = 0.05, one way ANOVA).

## Exploration of unexpected drug-drug relations

We explored the unexpected drug-drug relations regardless of the significance level. The TS and activity resemblance matrixes were computed and shown side by side for observation (**Figure S5**). The blocks in the activity matrix which were not in the TS matrix might indicate drug new applications or side effects. The drug indexes in the matrixes can be found in **Table S2**. To find interesting drug pairs with unexpected relations, one can quickly locate such drugs in the drug index table.

## References

1. The Anatomical Therapeutic Chemical (ATC) classification [http://www.whocc.no/atcddd/]
2. Kuhn M, Campillos M, Letunic I, Jensen LJ & Bork P (2010) A side effect resource to capture phenotypic effects of drugs. Mol Syst Biol 6: 343.
