# Supplementary figures and images for: Network-Based Relating Pharmacological and Genomic Spaces for Drug Target Identification
Source: PLoS One. 2010 Jul 26;5(7):e11764. doi: 10.1371/journal.pone.0011764 (PMC2909904; doi:10.1371/journal.pone.0011764)

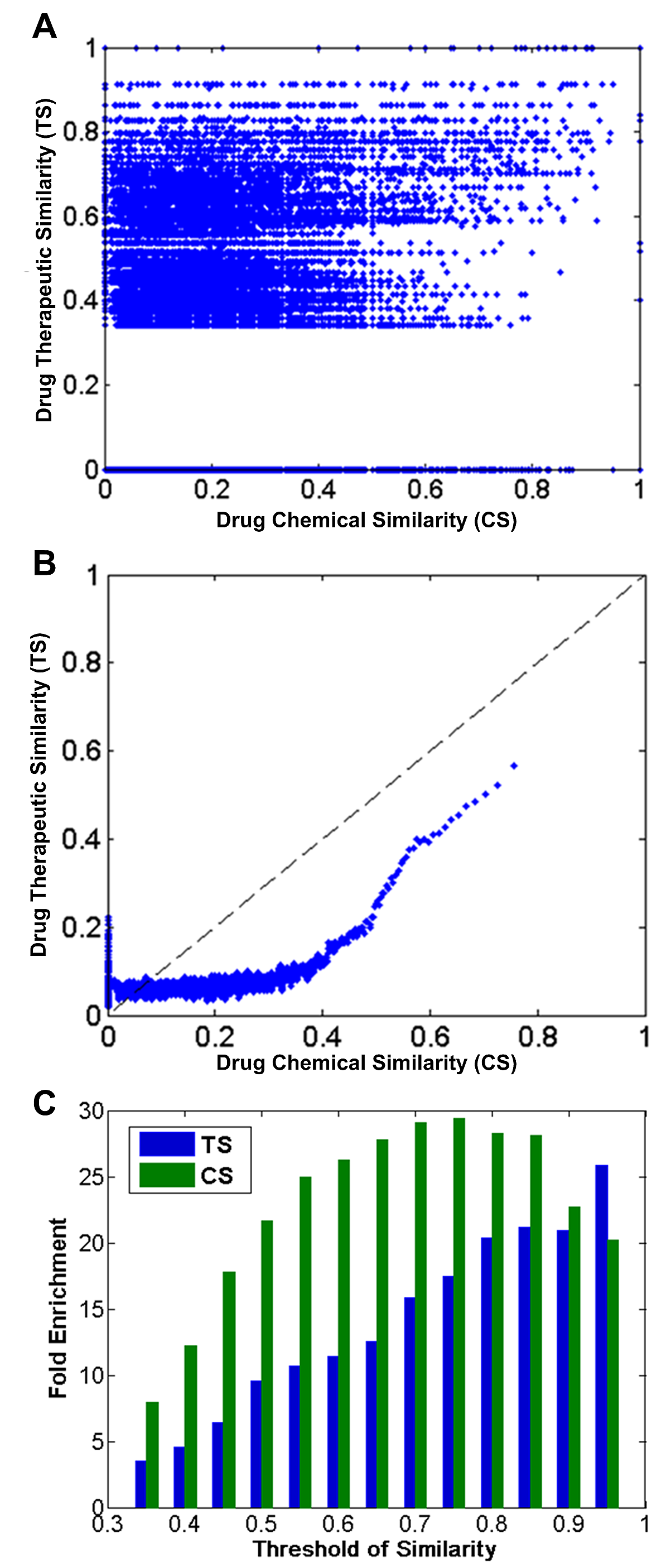

Supplement: Figure S1 — (a) Relationship between drug therapeutic similarity and chemical similarity. (b) Smoothed associations between drug therapeutic similarity and chemical similarity. (c) Fold enrichment analysis of therapeutic similarity and chemical similarity with respect to common target drug pairs. (10.15 MB TIF) [file pone.0011764.s004.tif]

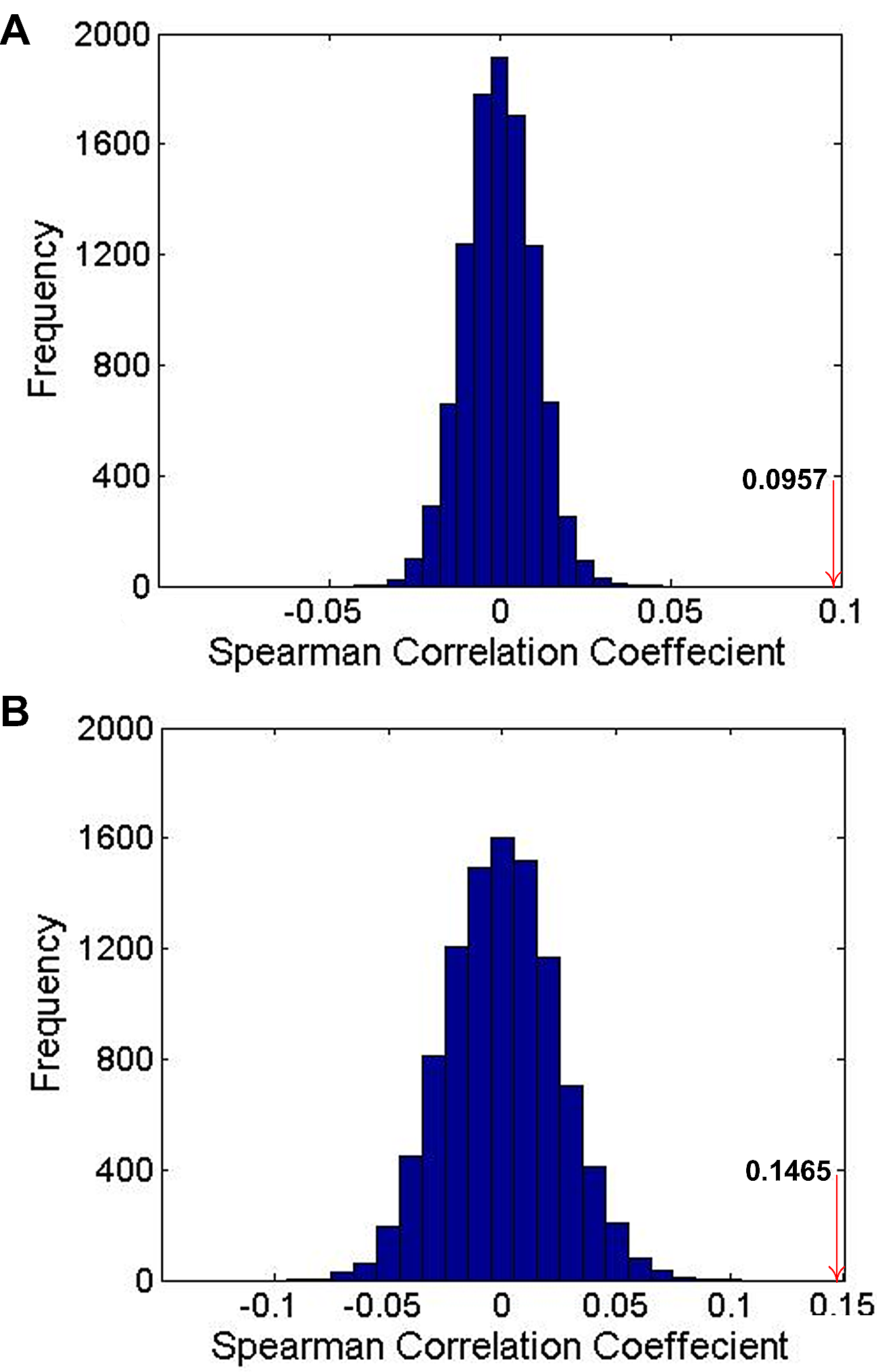

Supplement: Figure S2 — (a) Permuted correlation coeffecients for therapeutic similarity and dug genomic relatedness. (b) Permuted correlation coefficients for chemical similarity and drug genomic relatedness. (7.95 MB TIF) [file pone.0011764.s005.tif]

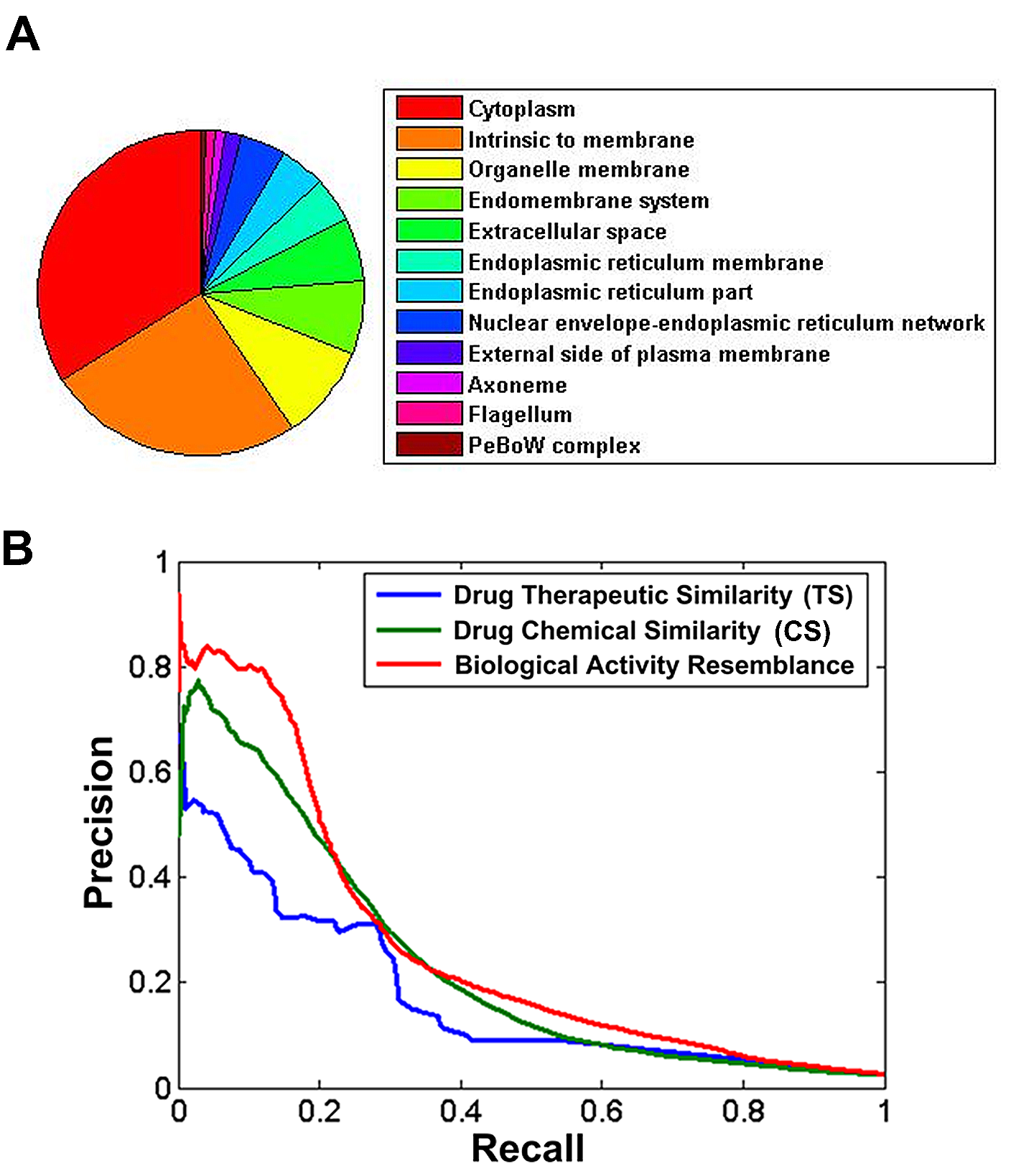

Supplement: Figure S3 — (a) The GO annotations (cellular component) for eliminated proteins. (b) Precision-Recall curves in recovering drug pairs with common targets. (6.97 MB TIF) [file pone.0011764.s006.tif]

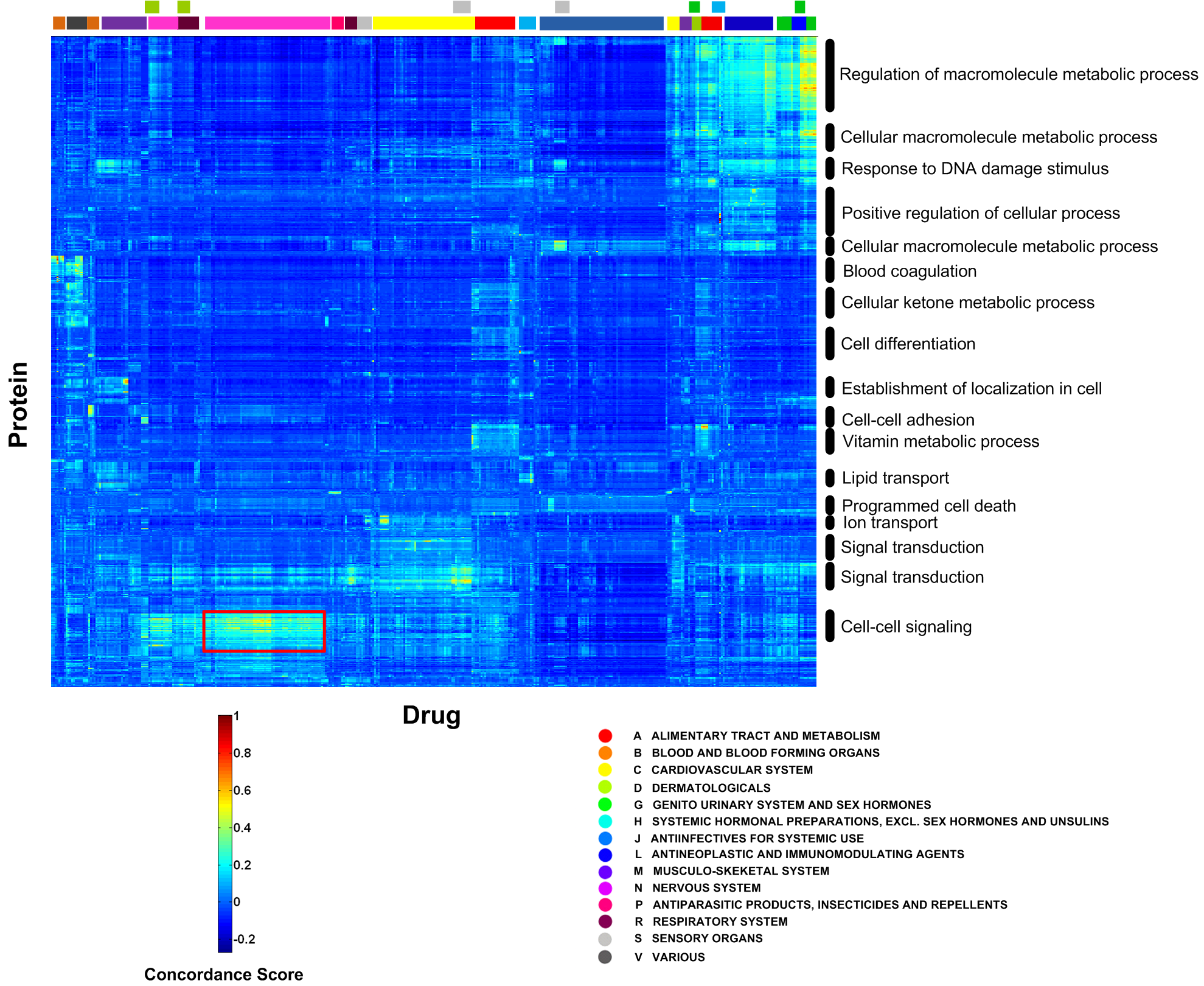

Supplement: Figure S4 — Two-way cluster for drug biological fingerprints. (9.62 MB TIF) [file pone.0011764.s007.tif]

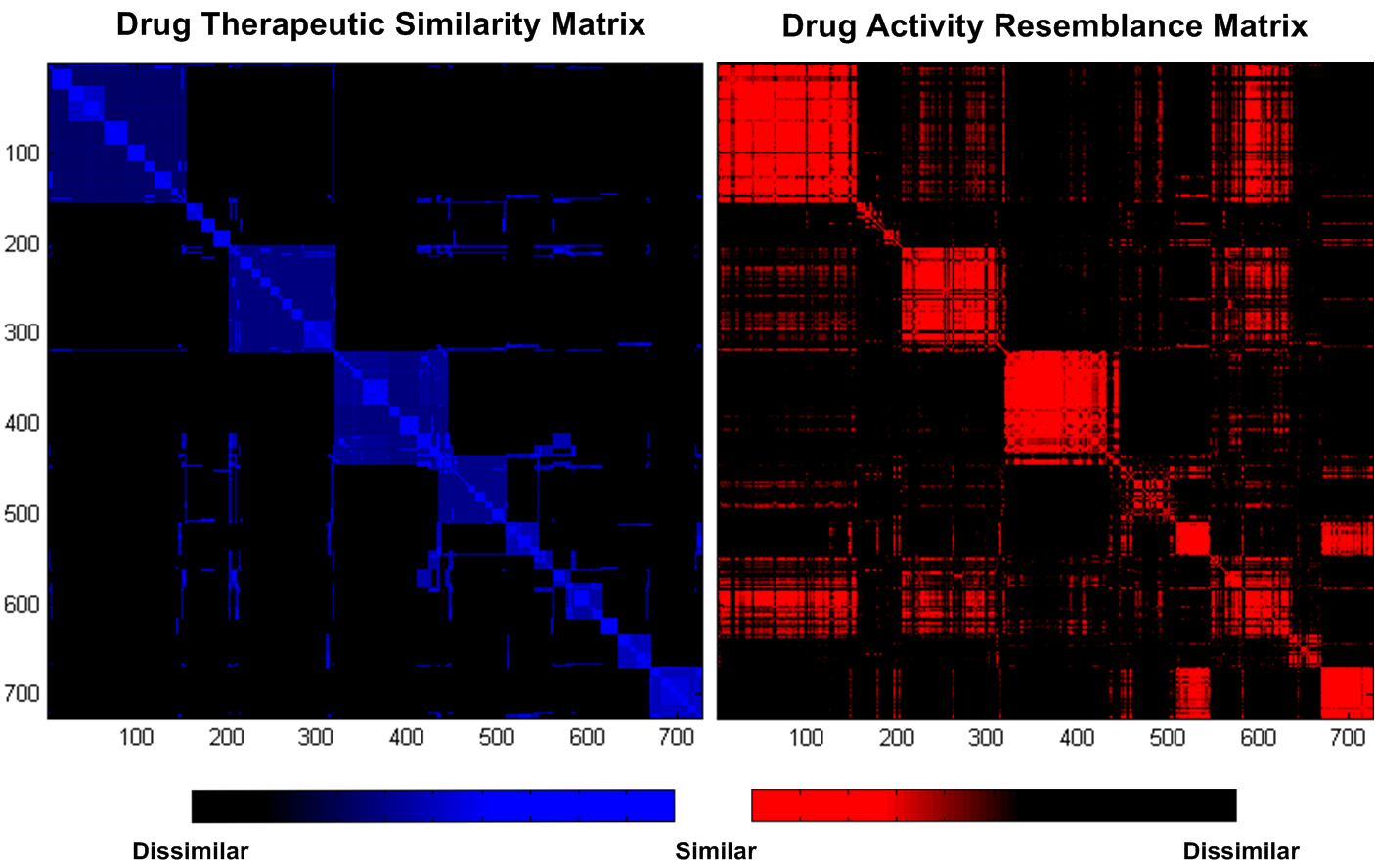

Supplement: Figure S5 — Comparison of drug therapeutic similarity and activity resemblance for unexpected drug-drug relations. (3.75 MB TIF) [file pone.0011764.s008.tif]
